# Label-free, rapid and quantitative phenotyping of stress response in *E. coli* via ramanome

**DOI:** 10.1038/srep34359

**Published:** 2016-10-19

**Authors:** Lin Teng, Xian Wang, Xiaojun Wang, Honglei Gou, Lihui Ren, Tingting Wang, Yun Wang, Yuetong Ji, Wei E. Huang, Jian Xu

**Affiliations:** 1 Single-Cell Center, CAS Key Laboratory of Biofuels and Shandong Key Laboratory of Energy Genetics, Qingdao Institute of BioEnergy and Bioprocess Technology, Chinese Academy of Sciences, Qingdao, Shandong, China; 2 University of Chinese Academy of Sciences, Beijing 100049, China; 3 Department of Engineering, University of Oxford, Oxford, Parks Road, OX1 3PJ, UK

## Abstract

Rapid profiling of stress-response at single-cell resolution yet in a label-free, non-disruptive and mechanism-specific manner can lead to many new applications. We propose a single-cell-level biochemical fingerprinting approach named “ramanome”, which is the collection of Single-cell Raman Spectra (SCRS) from a number of cells randomly selected from an isogenic population at a given time and condition, to rapidly and quantitatively detect and characterize stress responses of cellular population. SCRS of *Escherichia coli* cells are sensitive to both exposure time (eight time points) and dosage (six doses) of ethanol, with detection time as early as 5 min and discrimination rate of either factor over 80%. Moreover, the ramanomes upon six chemical compounds from three categories, including antibiotics of ampicillin and kanamycin, alcohols of ethanol and n-butanol and heavy metals of Cu^2+^ and Cr^6+^, were analyzed and 31 marker Raman bands were revealed which distinguish stress-responses via cytotoxicity mechanism and variation of inter-cellular heterogeneity. Furthermore, specificity, reproducibility and mechanistic basis of ramanome were validated by tracking stress-induced dynamics of metabolites and by contrasting between cells with and without genes that convey stress resistance. Thus ramanome enables rapid prediction and mechanism-based screening of cytotoxicity and stress-response programs at single-cell resolution.

Detection and characterization of stress-response in a cellular population or consortium have found numerous applications in life science and biotechnology industry^1^. Analysis of such response at the single-cell level is often advantageous or even essential, due to the ability to reduce cultivation time, to tackle yet-to-culture microbes and to distinguish genetically identical cells that exhibit biologically meaningful phenotypic difference^2^ (which can be crucial for adaptation to a fluctuating environment^3^).

A variety of such single-cell bio-sensing techniques, which detect and profile molecular information within individual cells, have been proposed^4^,^5^. A traditional approach is based on fluorescence labeling. For example, *E. coli* whole-cell biosensors, which carry a fluorescent protein-encoding reporter gene fused to a promoter inducible by particular stress, can be highly sensitive and specific, however the requirement for stress-specific promoters and transformability of cells limits their wider application. Moreover, as typically only one or a few molecules are labelled at a time, a comprehensive, mechanism-based view of cellular response is usually not possible. On the other hand, omics-based methods that profile molecules (e.g. mRNA^6^ and metabolites^2^) in individual cells can provide a landscape-like view of stress response, however the disruptive nature of such methods usually precludes rapid detection and sensing. Moreover, their application can be limited by the inability to amplify single-cell proteome and metabolome, potential bias associated with nucleic-acid amplification and typically high demand for consumable costs and technical skills. Therefore, a label-free, non-disruptive, simple yet sensitive method that rapidly yields a comprehensive, landscape-like profile of stress response program at single-cell resolution is of great value.

A Single-cell Raman Spectrum (SCRS) is a phenotypic profile of a single cell and can be obtained by Raman microspectroscopy (which directly detects vibrations of chemical bonds through the inelastic scattering by a laser light^7^) from an individual live cell in a label-free, non-disruptive manner. A SCRS sums up molecular vibrational signals in a single cell and provides a landscape-like profile of the cell^8^,^9^. SCRS has shown promise in identification of bacterial cells^10^,^11^,^12^, measurement of metabolite production^13^,^14^, and probing metabolic states of cells^15^,^16^,^17^,^18^,^19^ (see reviews^20^,^21^). We demonstrated the ability of SCRS to discriminate microalgal cells between nitrogen-replete and nitrogen-depleted conditions at 6 hours upon stress with >93.3% accuracy, and among the eight time points (from 0 hr to 96 hr) under stress with >90.4% accuracy^13^. One recent study showed that the temporal variation of *E. coli* SCRS was correlated with phenotypic response to the stress of 1.2%v/v n-butanol^22^. A second study suggested SCRS of *E. coli* can distinguish between individual antibiotics upon 30 min stress^19^. These observations usher in the hypothesis of whether SCRS in a cellular population can be exploited to detect, distinguish and characterize stress responses in a sensitive, specific and rapid manner. However, it is not known, (*i*) how SCRS is quantitatively influenced by the type, dose and duration of stresses; (*ii*) whether and to what degree SCRS can rapidly and specifically distinguish or reveal stress-response mechanisms among different classes of stressors; (*iii*) whether such cellular response as captured by SCRS is specific to a particular stress.

Here, employing *E. coli* as a model, we first showed that a “ramanome”, defined as the collection of SCRS from a number of cells randomly selected from an isogenic population at a given time and condition, is sensitive to both exposure time and dosage of ethanol, with detection time as early as 5 min (under 5%v/v Eth) and classification rate of either factor >80%. Moreover, ramanomes upon six chemical stressors from three categories, i.e., antibiotics of ampicillin (Amp) and kanamycin (Kan), alcohols of ethanol (Eth) and n-butanol (n-But) and heavy metals of Cu^2+^ (CuSO_4_) and Cr^6+^ (K_2_CrO_4_), were compared. A subset of 31 marker Raman bands representing a wide spectrum of chemical classes were revealed, which distinguished the cell responses via cytotoxicity mechanism. Furthermore, specificity, reproducibility and mechanistic basis of the method were validated by tracking stress-induced ramanomes between cells with and without genes that convey resistance to a particular antibiotic stress. The results suggested SCRS-based stress-response profiling can be label-free, rapid, quantitative, specific and mechanism-based, thus this approach is valuable to the screening of cytotoxicity, stress-response or stressors among individual prokaryotic or eukaryotic cells.

## Results and Discussion

**Figure 1:**
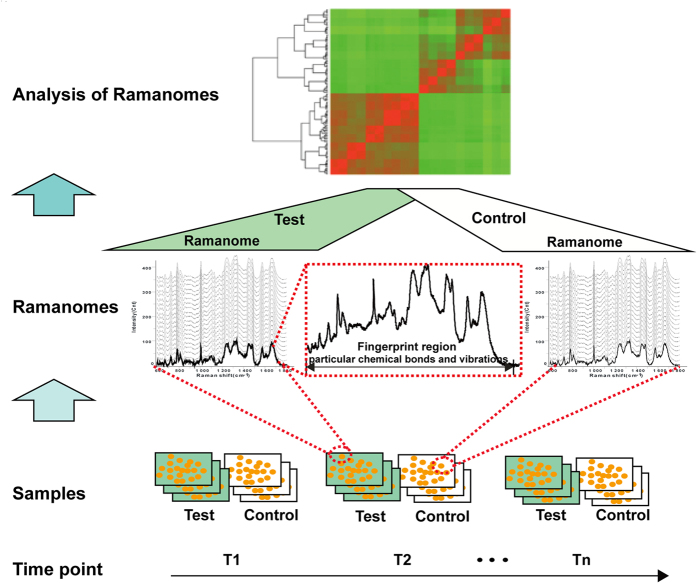
The definition of a ramanome. With a Raman band being conceptionally equivalent to a transcript or metabolite, a ramanome of a cell population provides a multiplex and comprehensive metabolic profile at not just the population level but the single-cell level.

As each of the 1581 Raman bands in a SCRS represents a particular type of molecular bonds in the cell, a SCRS can be considered as a digital image of thousands of pixels that carry the signature of the metabolome state of a cell. Comparison of the matching pixels between or among different time points or conditions thus can reveal features of the stress response program (Fig. 1). One “ramanome”, i.e., the collection of SCRS from multiple cells (e.g., 20) that were randomly sampled from the population at a given time and condition, was generated from each biological replicate of cell population (Fig. 1; Figure S-1B; Methods). To test whether and how the state of cellular response conveyed via ramanome is influenced by the type, duration and concentration of stressors, ramanomes under various duration or doses of Eth were first generated, and then time-series ramanomes were compared among six singular chemical stressors (Figure S-1B).

### Dose and duration sensitivity of ramanomes to ethanol

#### Dose-dependent ramanome variation

Stress-dose response of *E. coli* cells as measured by ramanome was profiled under the six Eth concentrations of 0% (control), 0.5%, 1%, 2%, 3% and 5% (v/v) after 30 min stress-exposure. Standard deviation of the mean (SDM)^12^,^13^ among biological replicates ranged from 0.12 ± 0.007 to 0.16 ± 0.005, supporting high reproductively of the ramanome measurement.

Significant distinction was detected between each of the five stressed ramanomes and the control (*p* = 0.001 for each; ANOSIM). For example, in discriminating between 0.5% (v/v) Eth and control, >95% accuracy was reached at 30 min, although the two growth curves were not significantly different even after 8 hr (Figure S-3A). Moreover, distance between the stressed and the control (as measured by R-value) is highly correlated with the dose (ANOSIM; Pearson coefficients *R*^2^ = 0.94; Table S-1), suggesting the ability of ramanome to model dosage response. In fact, the ramanome discriminated the five Eth doses from 0.5% to 5% with ~85% accuracy on average at 30 min, which is much more sensitive than OD_600_, as the latter failed to discriminate between 0.5% and 1% within 8 hr; Table S-1; Figure S-3A).

To identify dose-sensitive features, the stressed ramanomes were compared to the control via the fingerprint region of 640~1800 cm^−1^ (Figure S-2A). The results revealed high correlation of PC-LDA factor1 with dose (*R*^2^ = 0.88), supporting dose as main driver of ramanome variation (Figure S-2B). Dose-sensitive changes of SCRS were detected in 18 bands (revealed via PC-LDA factor1 between Eth-treated and Eth-free cells): e.g., intensity of nucleic-acid bands (1574, 1481, 811, 782, 728 cm^−1^, etc) was reduced dramatically with increasing Eth dose, while most protein (1002, 1242, 1308 cm^−1^, etc) and lipid (1661, 1448, 1127 cm^−1^, etc) bands exhibit an increase trend (within 30 min). These Eth-dose-sensitive bands can serve as individual markers in place of the whole fingerprint region (consisting of more than 1000 bands) for modeling dosage response.

#### Duration-dependent variation of ramanome

To test its sensitivity to stress duration, ramanome was measured after cells were stressed with 5% (v/v) Eth for 0 min, 5 min, 10 min, 20 min, 30 min, 1 hr, 3 hr, 5 hr, 8 hr and 20 hr, respectively. SDM among biological replicates ranged from 0.13 ± 0.003 to 0.17 ± 0.006, supporting technical reproducibility (Table S-2). Significant distinction was detected between a stressed ramanome and its corresponding control at each of the time points that started with 5 min (*p* = 0.001 for each; ANOSIM), suggesting rapid response of ramanome to stress. Moreover, distance between the stressed and the control followed a linear relationship with duration within the first 1 hr of Eth stress (*R*^2^ = 0.91; ANOSIM; Table S-2). Furthermore, ramanome is able to distinguish among each of the time points (and its associated condition) from 5 min to 20 hr, with 90% specificity and 90% sensitivity (Random Forest; Table S-2), supporting the value of ramanome in modeling the duration of stress. Interestingly, ramanome reaches ~94% sensitivity in distinguishing Eth-stressed cells at 5 min of exposure, as compared to 20 min using growth curve (tracked via OD600; Table S-2; Figure S-3B). This underscores the potential of ramanome in applications where rapid detection of stress response is required.

**Figure 2:**
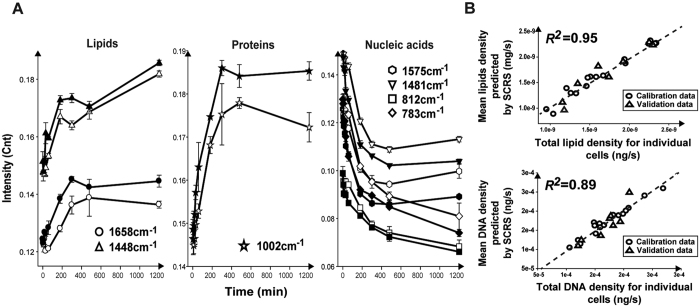
Tracking of *E. coli* ramanome during response to ethanol. (**A**) Temporal changes of Raman band intensity under control (empty symbol) or Eth-stress (solid symbol). Error bar represents SD (n = ~60). (**B**) PLSR model for quantification of lipid and DNA density in individual cells using ramanome. The mean lipid and mean DNA density of individual cells as predicted by ramanome was plotted versus the corresponding mean density determined by conventional methods (Methods). The unit of mg/s (ng/s) represents total mg (ng) biomass for each unit area of an individual cell.

Duration-sensitive Raman bands that serve as individual markers for predicting the temporal/stressed stage of a cell were identified via Random Forest, which consist of seven major nucleic-acid-, protein-, lipid-associated bands (derived from the first 50 Raman bands as ranked by significance). At either condition, intensity of such nucleic-acid bands (1575, 1481, 812, 783 cm^−1^, etc) exhibit gradual reduction temporally, while such protein (1002 cm^−1^, etc) and lipid (1658, 1448 cm^−1^, etc) bands exhibit gradual increase. Moreover, at each of the time points, intensity of the nucleic-acid bands in Eth-stressed cells was always lower than the control, yet the lipid bands were consistently higher (Fig. 2A). These observations collectively indicated that Eth altered the pace but not the program of physiological development.

#### Validation of Eth-induced phenotypic changes as captured by ramanome

To test whether the ramanomes provided mechanistic insights of stress response, density of intracellular nucleic acids and lipids were temporally profiled via conventional approach, in which metabolites were first extracted from culture-aliquots and then quantified (Methods; Supporting Information). To test the degree of correlation between the conventional and the ramanome-based approaches, a PLSR model^13^,^23^ was generated between averaged density of lipid/DNA in individual cells (conventional) and SCRS (ramaome-based), using the first two biological replicates from each approach as training data and the third as testing data. The correlation coefficients (*R*^2^) was 0.95 (0.97 for calibration dataset and 0.92 for validation dataset) for single-cell lipid density and 0.89 (0.96 for calibration dataset and 0.80 for validation dataset) for single-cell DNA density (Fig. 2B; Table S-3). These results underscored the ability of ramanome to provide insights into stress-response mechanisms.

### Variation of ramanome among different types and categories of chemical stressors

#### Analysis of ramanomes by ANOSIM and Random Forest

To probe whether ramanome can distinguish different stress-response programs, five additional chemicals from three categories were profiled for temporal dynamics (i.e., at seven time points within the first 5 hr of stress exposure, 5, 10, 20, 30, 60, 180, and 300 min), including a second alcohol of n-But, two antibiotics of Amp and Kan, and two heavy metals of Cu^2+^ and Cr^6+^. Doses of the chemicals were each set at a level that causes > 50% inhibition of growth within an 8 hr culture (Figure S-4A; Table S-4)^24^. SDM among biological replicates of ramanome ranges from 0.113 ± 0.009 to 0.168 ± 0.004 (the Pearson correlation is >0.98), suggesting high reproducibility of measurement (Table S-4).

**Figure 3:**
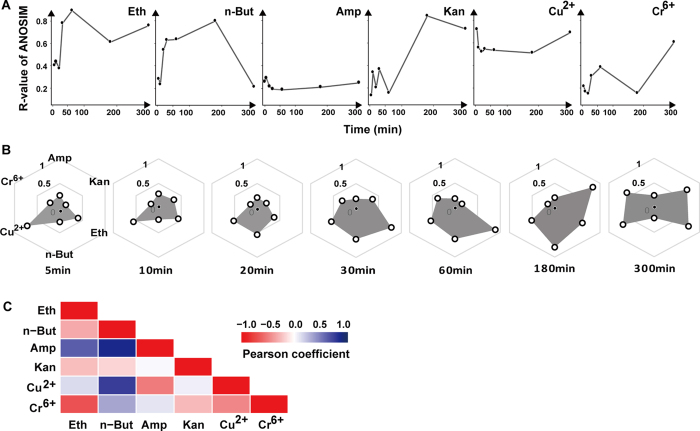
Comparison of ramanome between stressed and control cells. Similarity of ramanome was represented by R-value of ANOSIM. (**A**) Temporal tracking of ramanome variation under each stressor. (**B**) Radar map representing variation of ramanome among stressors at each time point. (**C**) Correlation coefficients (*R*^2^) of temporal patterns of ramanome among the stressors.

Significant distinction was detected between the stressed cells and their corresponding control cells at each duration time for each stressor (Figure S-5; *p* < 0.001). Moreover, temporal patterns of ramanome between stressed and control cells were highly diversified among the stressors (as represented by R-value; ANOSIM; Fig. 3A). The temporal pattern is relatively inert and stable for certain stressors (e.g. Amp), yet can be quite dynamic for others, exhibiting dramatic up-down (e.g., Eth and n-But), down-up (e.g., Cu^2+^) or more complex patterns (e.g., Kan and Cr^6+^; Fig. 3B). In fact, ramanome is able to reliably classify these stressors, as evidenced by 100% accuracy in classification (Random Forest) and the low Pearson correlation *R* values among the stressors (Fig. 3C).

#### Link and distinction among the six stress-response programs as unveiled by ramanome dynamics

**Figure 4:**
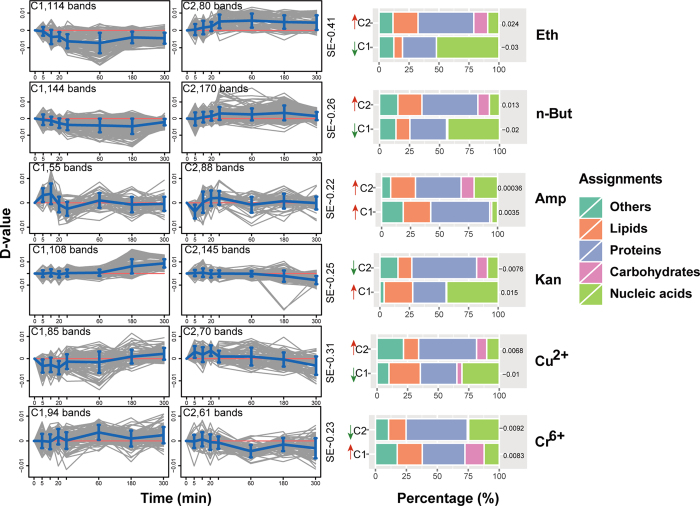
Temporal tracking of stress responses via marker Raman bands of each stressor. Temporal patterns of those Raman bands underlying each stress-response program were grouped into two clusters via K-means clustering based on the largest silhouette coefficient (SE). The change of mean difference value of Raman band intensity (D-value) between stressed and control cells in a given cluster was plotted as blue lines, with error bar representing SD (n = ~60). Values at 0 min were assumed as zero. Distribution of molecular functions as represented by the Raman bands within each cluster under each stressor was shown on the right.

To identify specific features that underlie the characteristic ramanome variation of each stressor, those Raman bands in the fingerprint region that exhibit significant variation between the stress and control conditions were first identified for each of the six stressors (Methods). They were then grouped into two clusters, C1 and C2, based on temporal variation patterns as compared to their corresponding controls for each stressor (K-means clustering with the largest silhouette coefficient or SE; Methods). In each cluster, the largest functional category annotated (accounting for 25~60%) is protein-related Raman bands^25^,^26^.

(*i*) Under Eth, 194 such bands were clustered into C1 (114 bands) and C2 (80 bands) (Fig. 4; SE = 0.41). In C1, ~50% Raman bands were assigned to nucleic acids, which underwent a gradual decrease after the onset of Eth. The significant decreased intensity of nucleic-acid bands under Eth was likely due to the “dilution” effect, as cell was greatly enlarged upon stress (1~6 fold longer and slightly wider; Figure S-4B), while there was no evidence for increased DNA/RNA degradation under Eth^24^,^27^,^28^,^29^. Cluster C2 featured increased intensities of lipid and carbohydrate bands, which was supported by enrichment of membrane-related biomass, such as membrane lipids and polysaccharides upon Eth stress^29^,^30^.

Under n-But (314 such bands, with 144 in C1 and 170 in C2; Fig. 4; SE = 0.26), for both clusters, composition and temporal features of bands were mostly similar to those under Eth. Transcriptomic and metabolic basis of cellular response under n-But stress were identical to those under Eth, especially in increase of carbohydrates^24^ and lipids^31^. However, Eth appears to induce a more dramatic change of ramanome (i.e., greater change in intensity of matching Raman bands). Moreover, lipid-related bands in C2 under n-But exhibit a temporal pattern distinct from those under Eth: at 30 min, lipids accumulated under Eth, yet without detectable change under n-But (Figure S-6). This led to distinct patterns of ramanome variation between the two alcohols (*p *< 0.001; Wilcoxon rank sum test).

(*ii*) Under Amp (143 such bands, with 55 in C1 and 88 in C2; Fig. 4, SE = 0.22), in C1, ~50% of the bands were assigned to proteins, whose intensities underwent an increase within 20 min of stress exposure (might result from the up-regulated amino-acids metabolism transcripts^32^), followed by a decrease afterwards until 1 hr (supported by the inhibition of certain protein metabolism transcripts under Amp, such as those of outer membrane protein biosynthesis^33^). In C2, bands assigned to carbohydrates were enriched as compared to C1; these bands underwent a decrease within 10 min and an increase to 1 hr, consistent with the induced expression of colanic acid (capsular exopolysaccharide of *E. coli*) biosynthetic pathway genes after 30 min of Amp exposure^33^, which helps cells cope with stress.

Under Kan (253 such bands, with 108 in C1 and 145 in C2; Fig. 4, SE = 0.25), nucleic-acid bands in C1 (~45% proportion) underwent an increase, which might be due to growth arrest rather than to direct response to Kan-stress. Extended duration of Kan-stress might result in retardation or pause of cell growth or reproduction (due to the inhibition of DNA-repair function by Kan^34^), resulting in temporal stability of nucleic acid level, which was in contrast to its temporal reduction in control cells. C2 featured a larger proportion of protein bands and the presence of carbohydrate bands. Kan functioned as an inhibitor of 30S ribosome and induced degradation of mistranslated proteins, and the resulted up-regulation of protease transcripts^35^ might explain the intensity decrease of proteins bands in C2. On the other hand, the relative signal reduction of carbohydrate bands in C2 (as compared to control) during Kan stress might be explained by the down-regulation of transcripts in cell wall biogenesis^32^.

(*iii*) Under Cu^2+^ (155 such bands, with 85 in C1 and 70 in C2; Fig. 4, SE = 0.31), in C1, nucleic acid and lipid bands were the second and third largest functional categories, showing an initial (within 1 hr) decrease followed by an increase after 3 hr of stress. The intensity reduction of nucleic-acid bands might be due to the slightly diluted density of cellular metabolites within 1 hr of stress caused by the enlarged cell size under Cu^2+^ (Figure S-4B), as well as due to DNA degradation^36^,^37^, while the increase after 3 hr of stress might indicate growth arrest (or cell death). For lipid-related bands, decrease in intensity of 1445 cm^−1^ (total lipids) and 1128 cm^−1^ (saturated lipids) was apparent during the first 1 hr of Cu^2+^ stress, suggesting high cellular sensitivity during this initial phase, as proportion of unsaturated fatty acids was found positively correlated with Cu^2+^ sensitivity^36^ (Fig. 4; Table S-5). However, the increased intensity of lipid bands after 5 hr stress duration might indicate loss of cell viability under Cu^2+^, which was consistent with the temporal pattern of nucleic-acid bands. Cluster C2, which is enriched in protein bands, experienced an increase within 30 min stress and then a decrease after 3 hr duration, which might be explained by the stimulation of genes encoding periplasmic proteins upon 5 min of Cu^2+^-stress^38^.

Under Cr^6+^ (155 such bands, with 94 in C1 and 61 in C2; Fig. 4, SE = 0.23), C1 exhibit a larger proportion of lipid and carbohydrate bands, as compared to C2; these bands underwent an increase trend, which was supported by up-regulation of transcripts in carbohydrate and lipid metabolisms under Cr^6+^ stress (e.g., in yeast^39^, Pseudomonas putida^40^,^40^ and Caulobacter crescentus^42^). C2 primarily featured decrease of protein bands under Cr^6+^, which was supported by the strongly inhibited expression of amino acid metabolism genes in *P. putida*^41^.

Between these two heavy metals, although the temporal patterns of ramanome are significantly different (*p* < 0.001; Wilcoxon rank sum test), those for the protein- and carbohydrate-related bands were mostly similar. This can be explained by the shared features of Cu^2+^- and Cr^6+^- responses such as enrichment of ribosome biogenesis and assembly genes (protein synthesis) and upregulation of common-metal-responsive genes in (carbohydrate metabolism)^39^.

### A Raman barcode distinguishes six stressors via mechanism of stress-response

#### Raman barcode of cellular-response to stress (RBCS)

By pooling those marker Raman bands that are both specific and shared among the six stress-response programs (Methods), we proposed RBCS, which consists of 31 elementary Raman bands that collectively characterize the temporal pattern of ramanome (Table S-5). Among them, six bands, representing nucleic acids (666, 811, and 1575 cm^−1^), proteins (853, 1302 cm^−1^) and lipids (957 cm^−1^) respectively, were found among the marker bands for all the six stressors, thus they correspond to a general cellular response. Between alcohols and heavy metals, the 13 shared bands included nine nucleic-acid bands (620, 720, 782, 823, 1092 and 1481 cm^−1^, in addition to the three all-shared nucleic acid bands above), thus changes of nucleic-acid appeared to be particularly responsive to alcohol and heavy metal stress. Between antibiotics and heavy metals, only seven bands were shared (including one protein band of 1620 cm^−1^, in addition to the six all-shared bands), indicating highly distinct mechanism of response.

Within the same stress category of alcohol, 18 of the 31 bands were shared between Eth and n-But, with most assigned to nucleic-acids and lipids (Table S-5). Fewer than a half of the bands (mostly lipids and protein related) were shared between Amp and Kan, suggesting a more different action mode. In contrast, 26 of the 31 bands (mostly protein assigned) were shared between Cu^2+^ and Cr^6+^, indicating a largely shared response mechanism to heavy metals.

**Figure 5:**
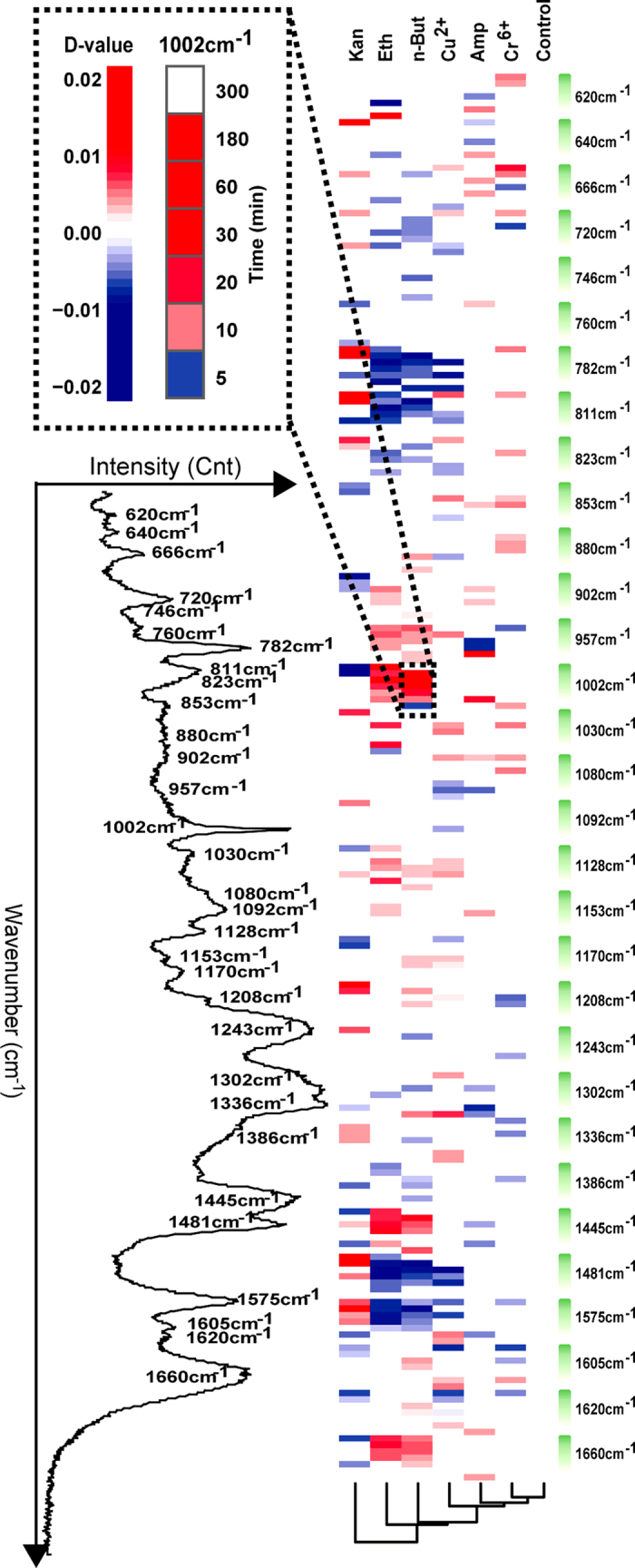
Raman-barcode of cellular-response to stressors (RBCS). The change in Raman band intensity was calculated as D-value (between stressed and control cells) and shown as blue (decreased intensity) or red (increased intensity) (*p* < 0.001; Wilcoxon rank sum test).

Clustering analysis of the RBCSs allowed mechanism-based classification of the six stress-response programs (Fig. 5). The clustering pattern of RBCSs was mostly consistent with that of the categories of alcohol, antibiotics and metals, except that the RBCSs of Amp and Kan were not clustered together (Fig. 5). These two antibiotics, with Amp from the β-lactam family and Kan from the aminoglycoside family, exert their anti-bacteria activity as cell-wall synthesis inhibitors and ribosome inhibitors respectively^34^. Kan stress inhibited protein synthesis and resulted in retardation or pause of cell growth, which was consistent with the decreased intensity of protein-related bands such as 853 and 1002 cm^−1^ and the increased intensity of nucleic-acid bands such as 782, 811, 1481 and 1575 cm^−1^ (as compared to control cells; Fig. 5). However, Amp mostly inhibits the synthesis of cell wall or cell membrane (consistent with the variation of lipid bands such as the up-regulated 1660 cm^−1^ and the down-regulated 957, 1302 and 1445 cm^−1^; Fig. 5). Thus clustering of RBCS is based on the stress-response mechanism, and not via the conventional classification scheme of chemicals.

#### Ramanome-based detection of cellular heterogeneity change under stress

**Figure 6:**
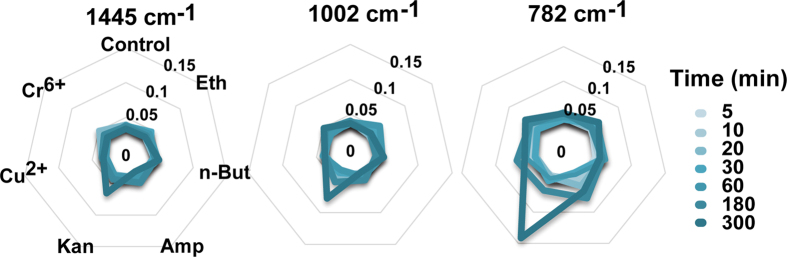
Comparisons of inter-cellular heterogeneity among stressors at each time point. Three Raman bands were shown as example, including 1445 cm^−1^ (lipids), 1002 cm^−1^ (proteins) and 782 cm^−1^ (nucleic acids). RSD, relative standard deviation.

In addition to stress-response phenotypes at the population level, ramanome provides temporal information for the degree of phenotypic heterogeneity among individual cells in a stress-response process. The relative standard deviation (RSD) of each of the Raman bands within a ramanome can be used to quantify the inter-cellular heterogeneity. For example, along the duration of stress-response, inter-cellular heterogeneity of the three member bands of RBCS, 1445 (Alkyl C-H_2_ bend, lipid), 1002 (benzene ring breathing, such as phenylalanine) and 782 cm^−1^ (cytosine, uracil, ring str, such as DNA/RNA) remained stable under alcohol-stressed, heavy-metal-stressed and control cells. However, the 782 cm^−1^ band underwent a remarkable increase upon 5 hr of antibiotics stress, particularly under Kan stress (*p* < 0.05, Kruskal-Wallis test; Fig. 6). On the other hand, Raman bands with different assignments can exhibit distinct heterogeneity patterns in response to environmental perturbation. For example, heterogeneity of the nucleic acid band 782 cm^−1^ underwent a sharp elevation specifically at 5 hr under Kan stress, likely due to the increased heterogeneity in growth stage among the cells as caused by Kan treatment.

### Specificity of the link between stress response and ramanome

To test the specificity of links between stress response and ramanome, the stress response programs as captured by ramanome were compared between wild-type *E. coli* DH5α (i.e., Kan^s^, stress-sensitive cells with blank plasmid) and its engineered strain into which resistance to a particular chemical stressor was introduced (i.e., Kan^r^, stress-resistant cells that harbor a Kan-resistant plasmid). As expected, WT+Kan exhibit a profoundly distinct RBCS from WT+H_2_O and from WT+Eth (Figure S-7A). Moreover, RBCS of Kan^s^+Kan clustered with that of WT+Kan, indicating that introduction of a control plasmid left the Kan-sensitive response program largely intact (Figure S-7A). Interestingly, the RBCS of Kan^r^+Kan is highly distinct from Kan^s^+Kan, and actually clustered with WT+H_2_O, suggesting introduction of Kan-resistant plasmid to host cells converted the stress-response program from a highly Kan-sensitive one to a resistant one. RBCS of Kan^r^+H_2_O was more identical to Kan^r^+Kan than to WT+H_2_O (Figure S-7A). Thus the observed variation between Kan^r^+Kan and WT+H_2_O is due to presence of the plasmid, which highlights the sensitivity and specificity of ramanome to stressors.

The stress-dependent variation of inter-cellular phenotypic heterogeneity observed also supported the specificity of links between stress response and ramanome. The heterogeneity under Kan^s^+Kan is identical to WT+Kan (*p* > 0.05) yet distinct from WT+H_2_O (*p* < 0.05), supporting a change of inter-cellular heterogeneity as induced by Kan. Interestingly, the heterogeneity under Kan^r^+Kan (e.g. RSD of 782 cm^−1^; Figure S-7B) is highly distinct from Kan^s^+Kan (*p *< 0.05), while is identical to that under WT+H_2_O (*p *> 0.05), suggesting introduction of Kan-resistant plasmid converted the heterogeneity pattern from a highly Kan-sensitive one to a resistant one. Thus the variation of heterogeneity under Kan stress, such as that for nucleic acids (i.e., 782 cm^−1^), was also highly specific and reproducible.

## Conclusions

Here we proposed the ramanome approach for the detection of stress-induced temporal variation from a cell population using single-cell Raman spectra (SCRS). We showed that ramanome was able to detect, distinguish and characterize both population-level features and inter-cellular variation of stress responses in a sensitive, specific, rapid and quantitative manner. Thus ramanome can serve as a multiplex, phenotypic and high-resolution signature for a particular state of stress response in *E. coli*. Moreover, RBCS which is a barcode of temporal pattern of 31 Raman bands was proposed as a cytotoxicity mechanism based signature for a stress-response program.

Compared to other single-cell stress-response profiling methods such as morphological analysis^43^, fluorescence imaging based biosensing^4^,^5^ or transcriptomics^6^, ramanome can be advantageous. Ramanome, in a label-free, non-disruptive and simple manner, rapidly yields a comprehensive and landscape-like view of molecular events of stress response without the need for preexisting biomarkers. Moreover, ramanome can be used as cell sorting criteria and applied to Raman activated cell sorting (RACS), such as Raman-activated Cell Ejecting (RACE^44^) and Raman-activated Microfluidic Sorting (RAMS^45^,^46^). Subsequent genomics and transcriptomics after RACS can establish specific links between DNA sequence or gene expression and a ramanome. For a given cell population, integrated profiling of ramanome, genome, transcriptome and lipidome should allow investigation into the genomic or transcriptomic basis of particular stress-response states or microevolution programs at single-cell resolution.

The sensitivity and dependency of ramanome to stressor, host cell genotype as well as a multitude of environmental variables (e.g., nutrient repletion/depletion, dissolved oxygen tension, osmolarity, temperature, pH, etc) suggested a probably unlimited size and search space for such multivariate single-cell biochemical-fingerprint data. Thus at present, when the scope and depth of available ramanome data are limited while the procedure or parameters of data measurements are not yet standardized, the practical utility of ramanome as a universal signature of stress response remains a question. For example, at the present stage it is not clear to what degree the change of cell type impacts the choice of Raman markers underlying RBCS. However, this question can be addressed by comparing the time-series ramanome datasets derived from different types of cells that span various phylogenetic distances. On the other hand, as the ramanome approach can be extended to theoretically any kinds of host cells (regardless of cultivability) and any abiotic or biotic stressors, a database of reference ramanome or RBCS for the most common cells and stressors (e.g. antibacterial agents or drugs) might be warranted. It can serve as a valuable community resource that aids rapid annotation or screening of unknown cells, stressors and stress-response programs. Moreover, global analysis of such phenotypic data for the vast combination of cells and stressors might provide new insights into diversity and mechanisms of stress-response programs in nature.

## Materials and Methods

### Strains and growth conditions

Stress response of *E. coli* DH5α cells to each of the six chemical stressors was assessed with 60 replicates (20 cells from each of the three biological replicates of cell culture) sampled at different states of cell growth. The cell growth was measured by OD_600_ in a 96-well plate. Dose of each stressor was set based on that producing > 50% inhibition of the growth within 8 hours.

### Single-cell Raman Spectrometry and chemometrics analyses

Sample preparation and then SCRS acquisition followed a consistent procedure (Figure S-1A; Supporting Information). Briefly, cells from each sample were spread onto CaF_2_ slides for 532 nm Raman spectra measurement. All raw Raman spectra (600 cm^−1^ to 1800 cm^−1^) from a triplicate of samples were baseline-corrected and normalized using Labspec 5, followed by chemometrics analyses such as Principal Component Analysis (PCA) and Principal Component and Linear Discriminant Analysis (PC-LDA), Random Forest, and Analysis of Similarities (ANOSIM). The Raman bands with significant changes under each stress were clustered via temporal patterns using K-means clustering with Euclidean distance (highest silhouette coefficients; R 3.0.3) (see more details in Supporting Information).

### Determining cellular DNA and lipid density via independent methods

Total DNA was isolated from 1 ml aliquots of cell culture using DNeasy Blood & Tissue kit (Qiagen) and then quantified by Qubit^®^ 2.0. Total lipids were measured via biphasic chloroform/methanol/- water extractions^26^, followed by drying under N_2_ flux and weighting in precision electronic balance. DNA/lipid content in individual cells was estimated by dividing the total weight by the number of cells. PLSR model was used to test the correlation between ramanome and the DNA/lipid density of individual cells as estimated above.

Full description of methodology is provided in Supporting Information.

## Additional Information

**How to cite this article**: Teng, L. *et al*. Label-free, rapid and quantitative phenotyping of stress response in *E. coli* via ramanome. *Sci. Rep.* **6**, 34359; doi: 10.1038/srep34359 (2016).

## Supplementary Material

Supplementary Information
