# Supplementary material for: Label-free, rapid and quantitative phenotyping of stress response in E. coli via ramanome
Source: Sci Rep. 2016 Oct 19;6:34359. doi: 10.1038/srep34359 (PMC5069462; doi:10.1038/srep34359)
Supplement: Supplementary Information [file srep34359-s1.pdf]

Supporting Information

Label-free, rapid and quantitative phenotyping of stress response in *E. coli* via ramanome

Lin Teng[1,2], Xian Wang[1,2], Xiaojun Wang[1,2], Lihui Ren[1], Tingting Wang[1], Yun Wang[1], Yuetong Ji[1], Wei E. Huang[3,*], Jian Xu[1,*]

[1]Single-Cell Center, CAS Key Laboratory of Biofuels and Shandong Key Laboratory of Energy Genetics, Qingdao Institute of BioEnergy and Bioprocess Technology, Chinese Academy of Sciences, Qingdao, Shandong, China

[2]University of Chinese Academy of Sciences, Beijing 100049, China

[3]Department of Engineering, University of Oxford, Oxford, Parks Road, OX1 3PJ, UK

*Corresponding author: xujian@qibebt.ac.cn or wei.huang@eng.ox.ac.uk

SUPPLEMENTAL MATERIALS AND METHODS

Growth, cultivation and stress treatment of E. coli cells

Cell culture E. coli DH5α cells were recovered on LB agar plate (10g/L NaCl, 10g/L Tryptone, 5g/L Yeast extract, 1.5% agar) from -80℃ stock 16h prior to the experiments. A single *E. coli* colony was then inoculated into 2ml LB medium in a glass tube and incubated at T=37℃, f=130rpm in a shaking incubator for 12h.

Stress treatment Each independent stress experiment was performed by exposing bacterial cultures to the stressor when OD_{600} of cells reached ~0.3 (UV/Vis spectrophotometer; GeneQuantTM 1300). A total of six broth cultures for each stress experiment were independently grown for Single-Cell Raman Spectra (SCRS) measurement, including three with the stressor (treatment samples) and three without (control samples, where an equal volume of ddH_2O was added in place of the stressor). Samples were collected after cells were stressed for various durations (e.g., for 0, 5, 10, 20, 30, 60, 180 and 300min). Cell pellets were first centrifuged gently at 5,200rpm for 1~2min at room temperature to minimize damages. Each sample was washed three times and suspended in ddH_2O to remove media residual, and then loaded onto a clean CaF_2 slide and air dried [1,2].

Single-cell Raman Spectrometry

Raman spectra acquisition Raman spectra of individual cells were acquired using a 100× magnifying dry objective (NA=0.9, BX41, Olympus, UK) and a 532 nm Nd:YAG laser (Ventus, Laser Quantum Ltd, UK) [3,4]. The power out of the objective was approximately 25 mW, and the diameter of laser spot was ~0.7μm. The scattered photons were collected by a

Newton EMCCD (Andor, UK) utilizing a 1600×200 array of 16 μm pixels with thermoelectric cooling down to -70℃ for negligible dark current. Acquisition of each SCRS was completed within 10s with spectral resolution of 1 cm^{-1}. Twenty cells were measured for SCRS in each biological replicate of cell culture.

Preprocessing of SCRS data Pre-processing of raw SCRS data was performed with LabSpec 5 (HORIBA Scientific, France) [3]. The averaged background (three to five spectra acquired from the area of slide surrounding the cell) was subtracted from each Raman spectrum, and then the intensities of the spectra were normalized to the total area under the curve [5]. Spectra were cropped to a spectral region of interest ranging from 600 to 1800cm^{-1} for chemometrics analysis.

Determination of lipid and DNA content of individual cells

Total lipid of a 100ml culture of *E. coli* cells was measured via biphasic chloroform-methanol-water extraction and then weighted in a precision electronic balance. Total DNA content of a 1ml culture of *E. coli* cells was estimated by extraction using DNeasy Blood & Tissue kit (Qiagen, Germany) and then quantification using Qubit® 2.0. The number of cells in the corresponding culture was counted by blood cell counting plate (average from three times for each sample). Thus single-cell lipid or DNA content was estimated by dividing the total lipids/DNA content by the total number of cells.

The density of lipids (or DNA) within a single cell was estimated via dividing single-cell total lipid (or DNA) content by the area size of a cell. To derive the area size of individual cells, fluorescence images of cells that were stained with 0.1% AO for 5min were

photographed within two seconds and exposed for 15μs (OLYMPUS, Japan). Software ImageJ was then used to estimate the area size of individual cells on the images.

Chemometrics analysis

PLSR model building A PLSR model was constructed using the Ramanome data and the experimental data (e.g., lipids and DNA density) in Matlab R2010a [4]. By relating the two datasets of X (intensity of related bands of SCRS) and y (single-cell lipid and DNA density estimated via conventional approaches) by means of regression, PLSR performs a multivariate calibration in order to establish a linear model which enables the prediction of y from the measured dataset of X. In the regression process, decomposition of X is performed under the consideration of y in a simultaneous analysis of the two datasets [6]. Specifically, the lipid or nucleic acid related Raman bands of SCRS of each of the 20 cells from triplicates at 0.5, 1, 3 and 5h were averaged separately, generating data of 24 (12 from each control groups and 12 from treatment groups) combined Raman spectra as a matrix (designed as X). Among them two of the triplicates at each time point were randomly selected to form a training dataset for calibration of the model (Xc, n=16), and the rest was used as a test dataset for validation (Xv, n=8). Correspondingly, single-cell lipid and DNA density of each triplicate culture at each time point were measured, generating data of another 24 values as a vector (designated as y), also including the training dataset (yc, n=16) and the test dataset (yv, n=8). Firstly, the PLSR model was established using the Xc and the yc data. Secondly, the Xv data and the function of the established model were used to predict the yv value. The predicted yv value was compared with the measured yv value. The reliability of the PLSR model was

assessed by the squared correlation coefficient (R^2) between the predicted and the measured y values in both the training set and the test set.

Comparing ramanomes within and among stressors The normalized fingerprint regions of SCRS were firstly subjected to principle component analysis (PCA) and LDA analysis [7] (~30 principal components used) for discrimination. Based on cosine-distance matric [8], ANOSIM was used to evaluate the similarity of ramanome between the control and the test at each time point (999 permutations; R version 3.0.3), the result (R-value) of which reflects the degree of cellular response. This provides a way to test statistically whether there is significant difference between two or more groups of sampling units. ANOSIM analysis returns two important factors, R-value (ranging from -1~+1; based on the ranks of dissimilarity between within-group and between-group; greater value means greater dissimilarity between the control and the test) and p value (to answer whether the difference is significant).

Random Forest Analysis Random Forest model was used to classify SCRS under the different stress treatments via default parameters (R package "randomForest", ntree=5,000, using default mtry of sqrt(p) where p is the number of Raman bands) [9]. Rank lists of Raman bands in the order of "band importance" by Random Forests were determined over 50 iterations of the algorithm. Raman datasets were reordered based on the rank list and then used as the input data for calculating the minimum number (N_{min}) of Raman bands for discriminating between the control and the stressed cells via ROC (receiver operating characteristic) analysis based on the largest AUC (area under the ROC curve) [10]. The top N_{min} ranking bands that showed significant difference between the control and the stressed were

designated as the marker bands for each of the stressors (Wilcoxon rank sum test; $p<0.001$).

SUPPLEMENTARY TABLES AND FIGURES

Table S-1. Analysis of ramanomes in the Eth dose-dependency experiments by Random Forest. Spe, specificity; Sen, sensitivity. Moreover, analysis of similarity (ANOSIM) was performed between the control cells and the stressed cells under each of the Eth doses (for the stress duration of 30min). **, $p<0.01$.

Eth (%v/v)	0	0.5	1	2	3	5	Spe(%)	Sen(%)	ANOSIM
**0**	57	0	3	0	0	0	71.3	95.0	-
**0.5**	5	48	2	3	2	0	82.8	80.0	0.16**
**1**	10	8	39	2	1	0	86.7	65.0	0.07**
**2**	7	2	1	44	6	0	83.0	73.3	0.36**
**3**	1	0	0	4	55	0	85.9	91.7	0.43**
**5**	0	0	0	0	0	60	100.0	100.0	0.84**

Table S-2. Ramanome-based models for discriminating temporal phases of ethanol response. Spe, specificity; Sen, sensitivity. C, control cells; e, stressed cells; m, minute; h, hour. Standard Deviation of the Means (SDM) of SCRS per sample was calculated (ranging from 0.13 to 0.17). Low SDM numbers represent high reproducibility and high reliability of the dataset. Analysis of similarity (ANOSIM) was performed between the control cells and the stressed cells at each time point. ** represents $p<0.01$.

	5m_c	5m_e	10m_c	10m_e	20m_c	20m_e	30m_c	30m_e	60mc	60me	3h_c	3h_e	5h_c	5h_e	8h_c	8h_e	20h_c	20h_e	Spe(%)	Sen(%)	SDM	ANOSIM
**5m_c**	59	1	1	1	0	0	0	0	1	0	0	0	0	0	0	0	0	0	**86.8**	**93.7**	**0.15±0.008**	**0.32****
**5m_e**	0	66	0	0	1	3	0	0	0	0	0	0	0	0	0	0	0	0	**89.2**	**94.3**	**0.14±0.006**	
**10m_c**	5	2	54	1	2	0	0	0	0	0	0	0	0	0	0	0	0	0	**90.0**	**84.4**	**0.15±0.008**	**0.37****
**10m_e**	1	1	1	60	1	2	0	1	1	0	0	0	0	0	0	0	0	0	**90.9**	**88.2**	**0.14±0.005**	
**20m_c**	1	1	2	0	54	3	1	0	2	0	0	0	0	0	0	0	0	0	**91.5**	**84.4**	**0.14±0.003**	**0.39****
**20m_e**	0	0	1	0	0	72	0	0	0	1	0	0	0	0	0	0	0	0	**85.7**	**97.3**	**0.14±0.003**	
**30m_c**	1	0	1	0	0	0	63	0	0	0	0	0	0	0	0	0	0	0	**96.9**	**96.9**	**0.14±0.008**	**0.71****
**30m_e**	1	0	0	2	0	3	0	53	0	0	1	0	0	0	0	0	0	0	**98.1**	**88.3**	**0.14±0.002**	
**60mc**	0	3	0	1	1	1	0	0	54	1	0	0	0	0	0	0	0	0	**90.0**	**88.5**	**0.14±0.006**	**0.89****
**60me**	0	0	0	1	0	0	1	0	2	55	0	0	0	0	0	0	0	0	**96.5**	**93.2**	**0.14±0.006**	
**3h_c**	0	0	0	0	0	0	0	0	0	0	55	1	0	0	0	0	0	0	**94.8**	**98.2**	**0.15±0.002**	**0.83****
**3h_e**	0	0	0	0	0	0	0	0	0	0	0	56	0	0	0	0	0	0	**94.9**	**100.0**	**0.13±0.003**	
**5h_c**	0	0	0	0	0	0	0	0	0	0	1	0	58	0	0	0	0	0	**96.7**	**98.3**	**0.15±0.009**	**0.75****
**5h_e**	0	0	0	0	0	0	0	0	0	0	1	0	0	60	0	2	0	2	**96.8**	**92.3**	**0.13±0.006**	
**8h_c**	0	0	0	0	0	0	0	0	0	0	0	1	2	0	53	0	1	0	**96.4**	**93.0**	**0.15±0.004**	**0.93****
**8h_e**	0	0	0	0	0	0	0	0	0	0	0	0	0	0	0	62	0	1	**96.9**	**98.4**	**0.13±0.006**	
**20h_c**	0	0	0	0	0	0	0	0	0	0	0	0	0	0	2	0	48	0	**98.0**	**96.0**	**0.17±0.006**	**0.50****
**20h_e**	0	0	0	0	0	0	0	0	0	0	0	1	0	2	0	0	0	57	**95.0**	**95.0**	**0.14±0.007**	

Table S-3. Temporal variation of single-cell DNA and lipid density in the control cells and 5%-ethanol-stressed cells as determined by the conventional approaches. The density of DNA (lipids) of a cell was calculated as the ratio between the averaged total DNA (lipid) content of a cell and the averaged area of a single cell. Error bars represent standard errors (n≥3).

Group	Time (min)	Cell size ($inch^2$)	DNA		Lipids	
			Cell (10^9)	Density (10^{-4} ng*$inch^2$/cell)	Cell (10^{10})	Density (10^{-9} mg*$inch^2$/cell)
Control	30	0.092±0.021	1.49±0.03	2.39±0.30	1.74±0.07	1.05±0.09
	60	0.089±0.034	1.58±0.08	1.97±0.07	1.76±0.04	1.17±0.10
	180	0.047±0.013	1.67±0.02	1.91±0.16	3.29±0.20	1.53±0.07
	300	0.035±0.009	1.47±0.03	1.81±0.17	2.46±0.08	2.28±0.05
Ethanol	30	0.097±0.028	1.64±0.04	1.59±0.00	1.25±0.06	1.34±0.10
	60	0.109±0.041	2.44±0.01	1.58±0.14	1.08±0.06	1.67±0.08
	180	0.095±0.026	0.25±0.01	1.27±0.22	1.07±0.08	1.94±0.02
	300	0.108±0.044	0.21±0.02	1.22±0.03	0.74±0.02	2.30±0.04

Table S-4. The stressor, number of SCRS, standard deviation of the means (SDM) and Pearson correlation coefficient (*r*) of SCRS that are associated with each sample. Data represent mean values ± standard deviations (n = 60). C, control cells; T, stressed cells.

Groups		Time (min)	No. spectra	SDM	*r (%)*
ddH_2O	C	0	162	0.147±0.004	98.9±0.2
Amp stress (2mg/L, Solarbio)	C	5	60	0.133±0.005	99.1±0.1
	T		60	0.143±0.002	99.0±0.1
	C	10	56	0.146±0.007	98.9±0.2
	T		53	0.160±0.007	98.7±0.4
	C	20	60	0.138±0.002	99.0±0.2
	T		59	0.149±0.004	98.9±0.2
	C	30	62	0.138±0.006	99.0±0.2
	T		62	0.144±0.002	99.0±0.1

	C	60	61	0.135±0.011	99.1±0.2
	T		61	0.140±0.006	99.1±0.2
	C	180	60	0.132±0.005	99.1±0.1
	T		60	0.140±0.004	98.9±0.3
	C	300	60	0.134±0.007	99.0±0.2
	T		60	0.144±0.005	98.9±0.2
Kan stress (3.7mg/L, Solarbio)	C	5	58	0.143±0.011	98.9±0.2
	T		60	0.145±0.004	99.0±0.2
	C	10	59	0.150±0.006	98.8±0.2
	T		69	0.148±0.004	99.0±0.1
	C	20	60	0.138±0.007	99.0±0.2
	T		60	0.142±0.005	99.0±0.1
	C	30	62	0.130±0.003	99.1±0.1
	T		62	0.140±0.002	99.0±0.2
	C	60	60	0.127±0.002	99.2±0.1
	T		61	0.127±0.002	99.3±0.2
	C	180	60	0.132±0.005	99.1±0.1
	T		60	0.128±0.008	99.1±0.2
	C	300	60	0.138±0.007	99.0±0.2
	T		60	0.168±0.004	98.4±0.9
Eth stress (5%v/v, Sinopharm(Cp))	C	5	60	0.137±0.006	99.0±0.1
	T		60	0.137±0.004	99.2±0.2
	C	10	58	0.138±0.003	99.0±0.2
	T		66	0.143±0.001	99.1±0.2
	C	20	62	0.135±0.004	99.1±0.2
	T		68	0.139±0.004	99.1±0.2
	C	30	63	0.130±0.007	99.1±0.2
	T		60	0.142±0.001	99.1±0.2
	C	60	66	0.129±0.005	99.1±0.2
	T		60	0.145±0.007	99.0±0.2
	C	180	58	0.131±0.007	99.0±0.2
	T		65	0.132±0.011	99.2±0.2
	C	300	60	0.143±0.009	99.0±0.2
	T		61	0.141±0.008	99.3±0.2
n-But stress (0.8%v/v, Sinopharm(Cp))	C	5	69	0.133±0.004	99.1±0.1
	T		73	0.136±0.003	99.1±0.1
	C	10	71	0.135±0.008	99.0±0.2
	T		72	0.139±0.004	99.1±0.1
	C	20	70	0.137±0.003	99.0±0.1
	T		56	0.148±0.005	98.9±0.2
	C	30	67	0.130±0.001	99.1±0.1
	T		71	0.142±0.002	99.0±0.2
	C	60	70	0.131±0.007	99.1±0.2

	T		61	0.149±0.004	98.8±0.2
	C	180	50	0.139±0.010	99.0±0.2
	T		47	0.147±0.002	99.1±0.2
	C	300	71	0.113±0.009	99.3±0.1
	T		60	0.118±0.010	99.3±0.3
CuSO4 stress (3.8mM, Aldrich)	C	5	61	0.130±0.006	99.1±0.2
	T		60	0.136±0.002	99.2±0.2
	C	10	60	0.130±0.005	99.1±0.2
	T		60	0.131±0.006	99.3±0.2
	C	20	57	0.130±0.009	99.1±0.2
	T		60	0.130±0.004	99.3±0.1
	C	30	60	0.126±0.008	99.2±0.2
	T		60	0.127±0.005	99.3±0.1
	C	60	70	0.127±0.004	99.1±0.2
	T		60	0.132±0.001	99.2±0.1
	C	180	54	0.144±0.006	98.9±0.2
	T		60	0.144±0.003	99.2±0.2
	C	300	60	0.141±0.006	98.9±0.2
	T		60	0.147±0.003	99.1±0.2
K2CrO4 stress (0.2mM, Sinopharm (Ap))	C	5	60	0.135±0.003	99.0±0.2
	T		60	0.143±0.004	98.9±0.1
	C	10	60	0.141±0.008	99.0±0.2
	T		60	0.147±0.003	98.9±0.2
	C	20	60	0.137±0.004	99.0±0.2
	T		60	0.148±0.002	99.0±0.3
	C	30	60	0.135±0.003	99.0±0.1
	T		60	0.146±0.005	98.9±0.2
	C	60	58	0.127±0.004	99.1±0.2
	T		60	0.139±0.004	99.0±0.2
	C	180	54	0.144±0.006	98.9±0.2
	T		60	0.149±0.007	98.8±0.2
	C	300	60	0.141±0.006	98.9±0.2
	T		60	0.140±0.003	98.9±0.2

Table S-5. The 31 Raman bands that underlie RBCS. Raman bands that changed significantly were labeled as "•", while those of them that are shared among the six stressors were underlined.

Raman bands (cm^{-1})	Assignments	Role in stress response					
		Eth	n-But	Am	Ka	Cu^{2}	Cr^{6}
~620	C-C twisting mode of phenylalanine (proteins)	●	●	●		●	●
~640	C-S stretching & C-C twisting of proteins (proteins)	●		●	●		
**~666**	G, T (DNA/RNA), C-S stretching mode of cystine (proteins)	●	●	●	●	●	●
~720	DNA, characteristic for phospholipids (lipids)	●	●		●	●	●
~746	T (ring breathing mode of DNA /RNA bases)		●	●	●		
~760	Tryptophan, δ (ring) (proteins)			●	●		
~782	U, T, C (ring breathing modes in the DNA /RNA bases)	●	●		●	●	●
**~811**	C-C stretching (proteins), C_5' -O-P-O-C_3' phosphodiester (RNA)	●	●	●	●	●	●
~823	tyrosine (proteins), O-P-O stretch DNA	●	●		●	●	●
**~853**	tyrosine & C-C stretch of proline ring, (C-O-C) skeletal mode of α-anomers	●	●	●	●	●	●
~880	Tryptophan, δ (ring) (proteins)		●	●		●	●
~902	(C-O-C) skeletal mode	●	●	●	●		
**~957**	cholesterol	●	●	●	●	●	●
~1002	Phenylalanine, C-C skeletal (proteins)	●	●	●	●		●
~1030	δ(C-H), C-N stretching (proteins), υ(CO), υ(CC), υ(CCO) (polysaccharides)	●			●	●	●
~1080	Typical phospholipids (lipids), Phosphate vibrations, Collagen (proteins)		●	●		●	●
~1092	Symmetric phosphate stretching vibrations (DNA)	●	●		●	●	●
~1128	C-N stretching (proteins), C-O stretching (carbohydrates), υ(C-C) in lipid	●	●		●	●	
~1153	υ(C-N), proteins (proteins)	●		●			
~1170	C-H in-plane bending mode of tyrosine, (CH) phenylalanine (proteins)		●	●	●	●	
~1208	υ(C-C_6H_5), tryptophan, phenylalanine (proteins), A,T (DNA /RNA)		●		●	●	●
~1243	Amide III (proteins), Asymmetric phosphate (DNA/RNA)		●		●		●
**~1302**	CH_3 /CH_2 twisting or bending mode of lipid/collagen (lipids or proteins)	●	●	●	●	●	●
~1336	CH_3CH_2 deforming modes of collagen (proteins) and nucleic acids		●		●	●	●
~1386	CH_3 band (lipids)	●	●		●		●
~1445	CH_2 bending and scissoring modes of collagen and phospholipids	●	●	●	●		
~1481	Amide II (proteins), Nucleotide acid purine bases (DNA/RNA)	●	●		●	●	●
**~1575**	Ring breathing modes in the DNA bases (DNA/RNA)	●	●	●	●	●	●
~1605	Phenylalanine, tyrosine, C=C (proteins)		●		●	●	●
~1620	υ(C=C), tryptophan (proteins)		●	●	●	●	●
~1660	υ(C=C) cis, lipids, unsaturated fatty acids, Amide I (proteins)	●	●	●	●		

Figure S-1. Experimental design and workflow for generation of ramanome datasets. (**A**) Experimental work flow for a typical ramanome experiment. (**B**) Global design of this study. View I explored the effect of duration and doses on ramanome variation. View II investigated the impact of six stressors on ramanome. View III tested the specificity between ramanome and stress-response.

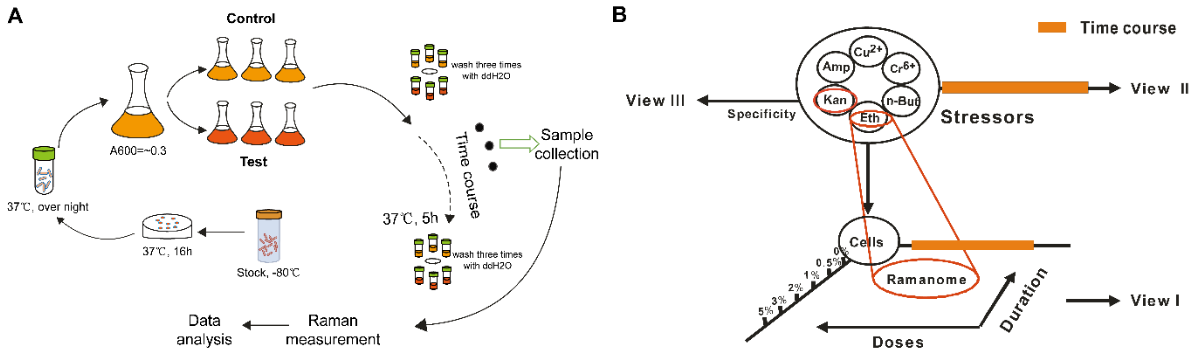

Figure S-2. PC-LDA analysis of cells cultivated under varied ethanol concentrations. (**A**) PC-LDA score plot for discriminating cells under different Eth concentrations. Each dot represents a cell. (**B**) Comparison of scores in the direction of PC-LDA factor1 (PC-LD1) among cells under the various Eth concentrations. Raman bands that discriminated cellular response among the Eth doses were colored either green (if band intensity was reduced under stress as compared to the control) or yellow (if band intensity was elevated under stress as compared to the control). Each triangle represents an average score (among 60 cells) of PC-LDA on the direction of factor1 at each time point.

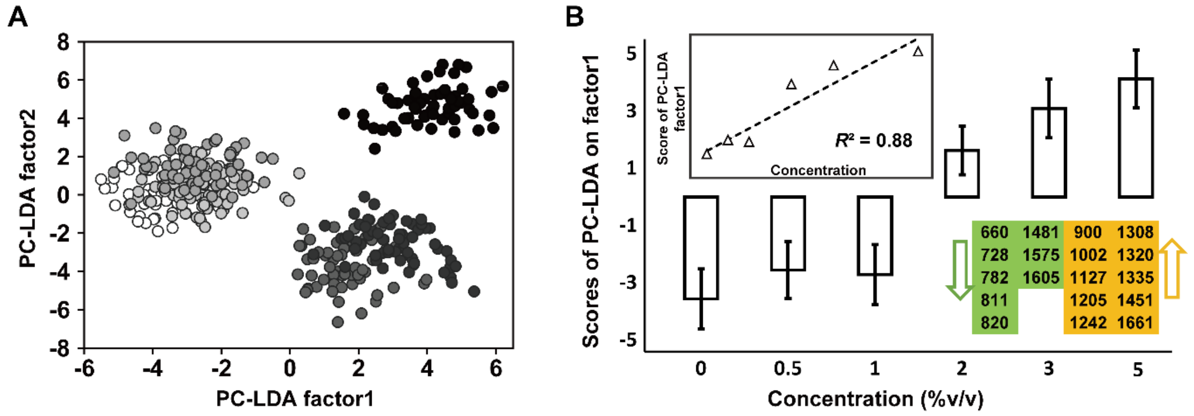

Figure S-3. Growth of *E. coli* DH5α cells under various ethanol concentrations as measured by OD600. (**A**) Growth curves under various doses of ethanol. Cells under 0.5% and 1% (v/v) ethanol showed no significant growth inhibition at 8hr as compared to the Eth-free condition (p>0.05; Student t-test). (**B**) Comparison of cell growth (as measured by OD600) within one hour of stress duration between the 5% (v/v) ethanol and the ethanol-free conditions . Eth, ethanol. Significant difference as indicated by Student's t-test is indicated as an asterisk. *, 0.01<p<0.05; **, p<0.01. Values represent means ±SD (n=3~4).

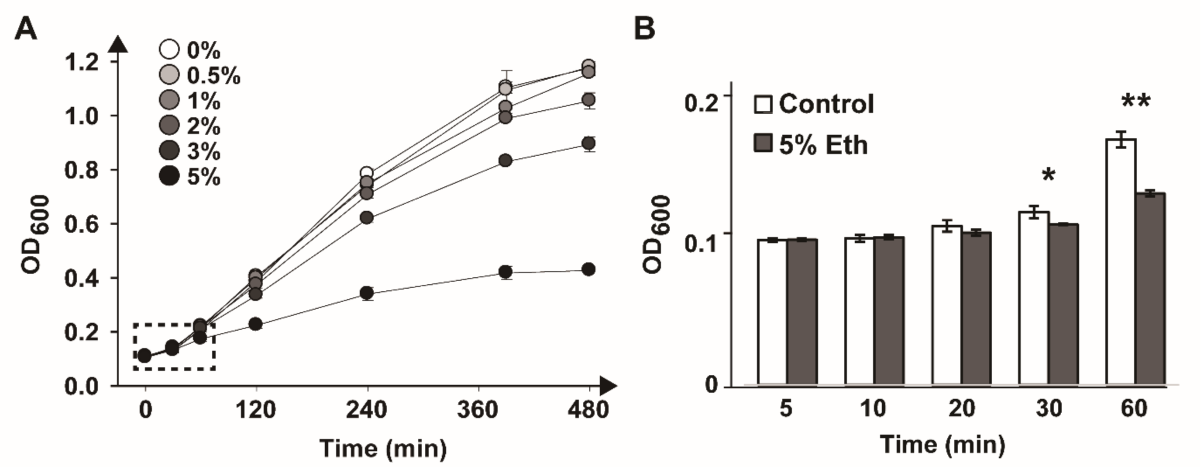

Figure S-4. Growth curve and morphological change of *E. coli* DH5α cells under the six stress conditions. (**A**) Growth curves as measured by OD600. (**B**) Comparison of cellular morphology between the control and the stressed cells at 1h and 5h of stress exposure respectively for each of the six stressors. Spatial scale (1μm) is labeled at bottom right corner of each image (100×). Eth, 5% (v/v) ethanol; n-But, 0.8%(v/v) n-butanol; Amp, 2mg/L ampicillin; Kan, 3.7mg/L kanamycin; Cu^{2+}, 3.8mM $CuSO_4$; Cr^{6+}, 0.2mM K_2CrO_4.

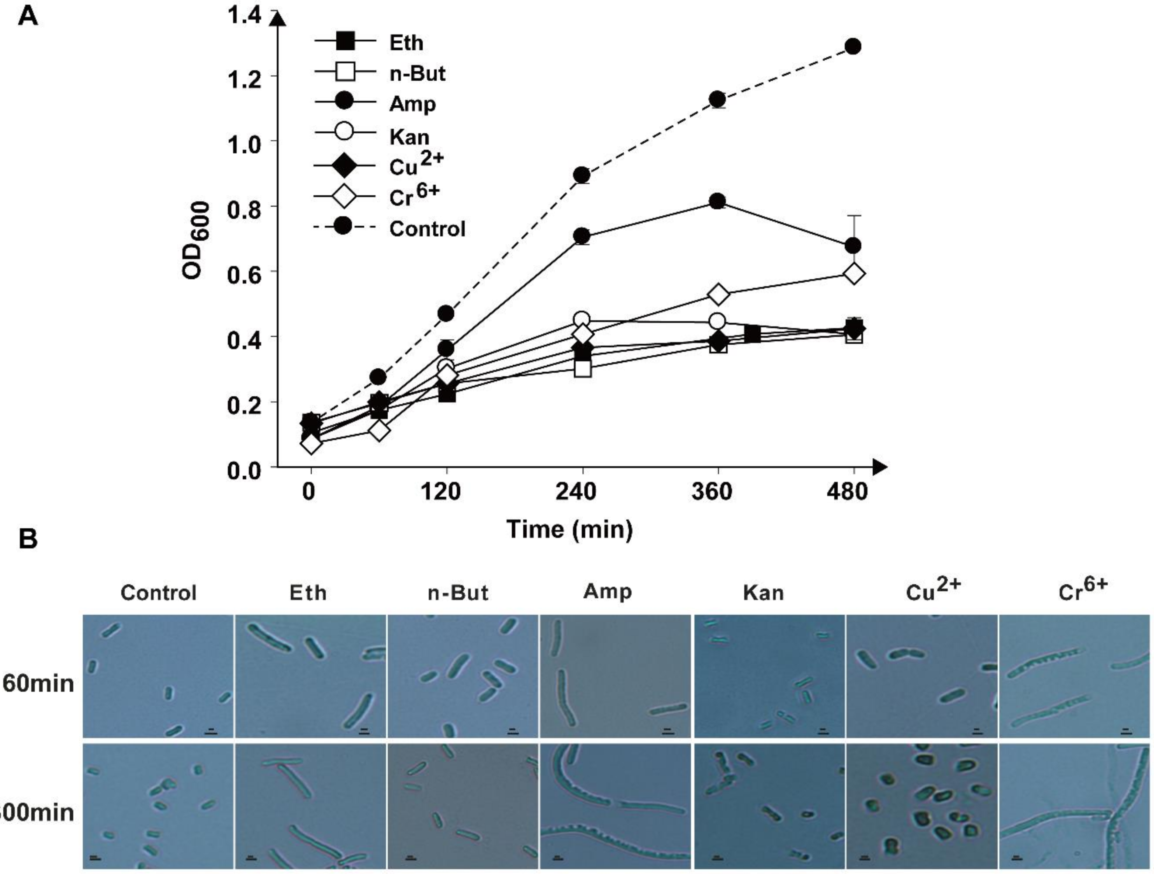

Figure S-5. Temporal tracking of cellular stress response via ramanome. PCA score plots were shown.

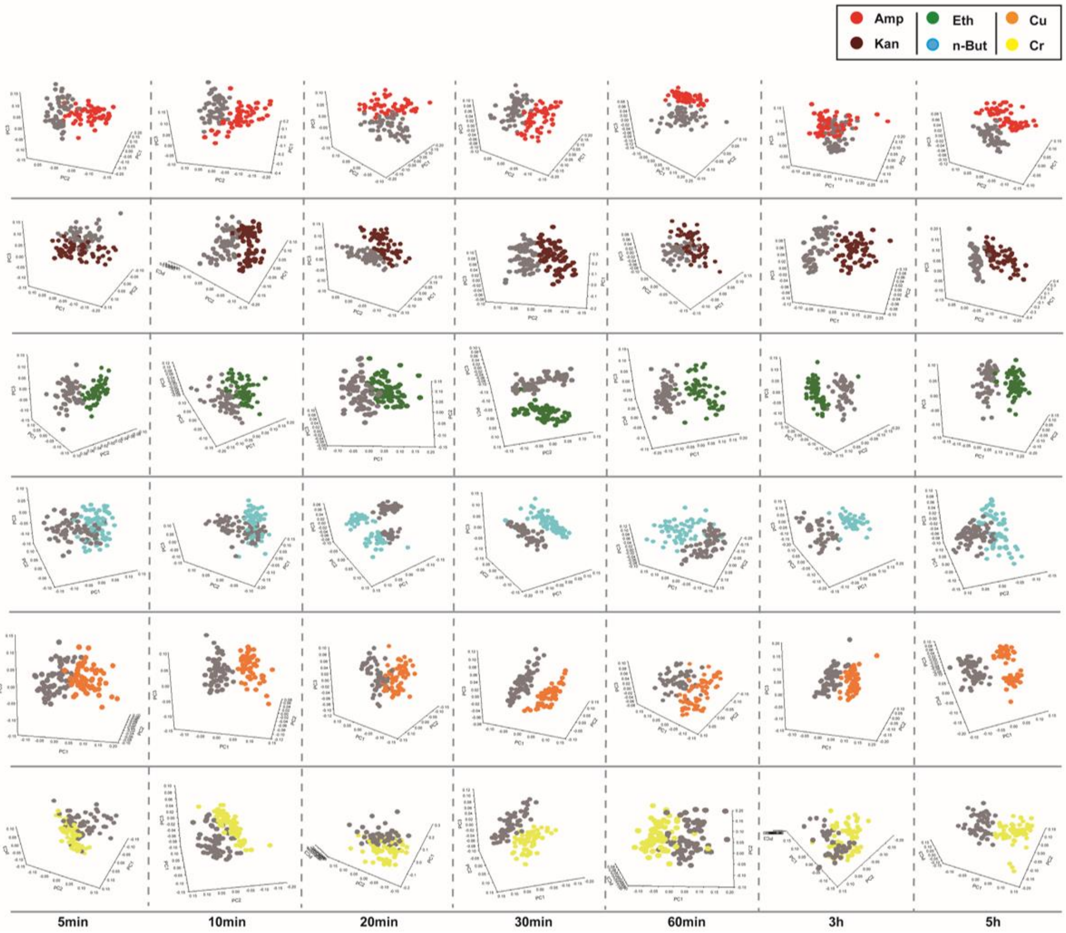

Figure S-6. Ramanome-based comparison of cellular stress response across the six stressors at each of the seven time points. For each stressor, the underlying marker Raman bands were clustered at each of the time points based on their variation as compared to the control (K-means clustering; largest silhouette coefficient). Shown on the right are the metabolite classes as represented by the Raman bands. The clusters in the left panel, shown in different colors and ordered from bottom to top (as indicated by the vertical block arrows) correspond to the clusters in the right panel (ordered from left to right; the horizontal block arrows).

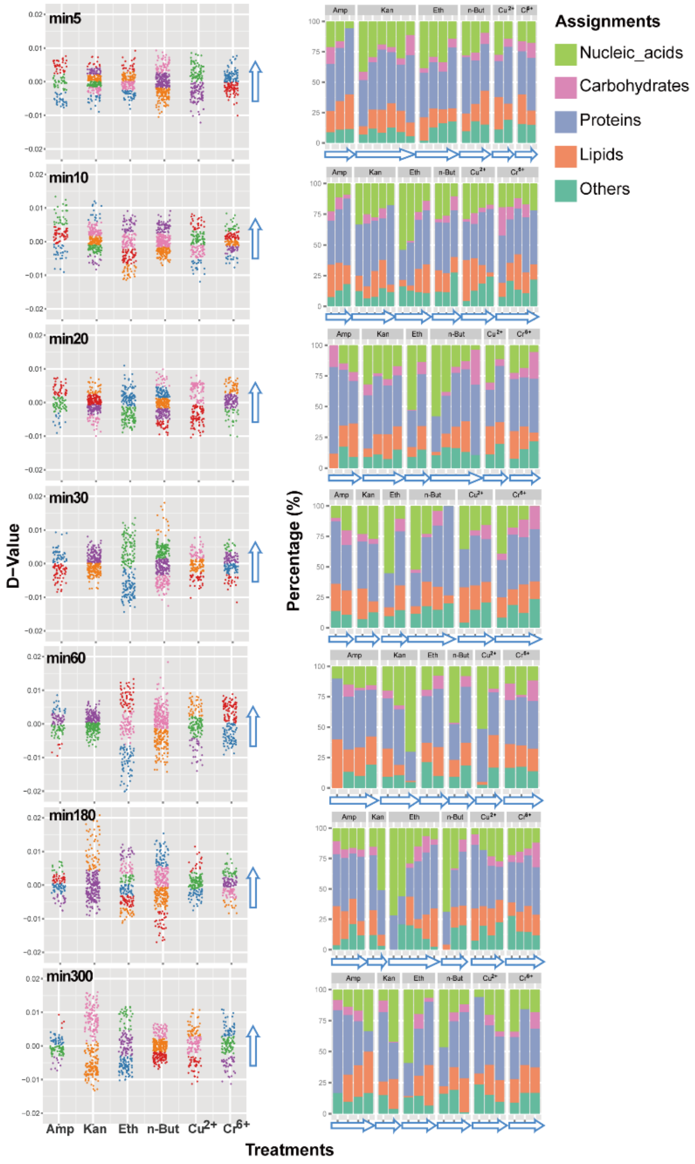

Figure S-7. Specificity of the link between RBCS and stress-response. (**A**) Specificity of Kan stress-response program captured by RBCS as revealed by hierarchical clustering of RBCS. (**B**) Specificity of heterogeneity under Kan stress captured by RBCS as represented by RSD for three Raman bands (1445cm^{-1}, 1002cm^{-1} and 782cm^{-1}). WT, control cells; Kan^{s}, engineered DH5α cells that harbor a control plasmid (i.e., without Kan-resistance gene); Kan^{r}, engineered DH5α cells that harbor a plasmid with Kan-resistance gene. Statistical significance of RSD difference: *, 0.01<p<0.05; **, p<0.01; "NS", p>0.05 (Student's t-test).

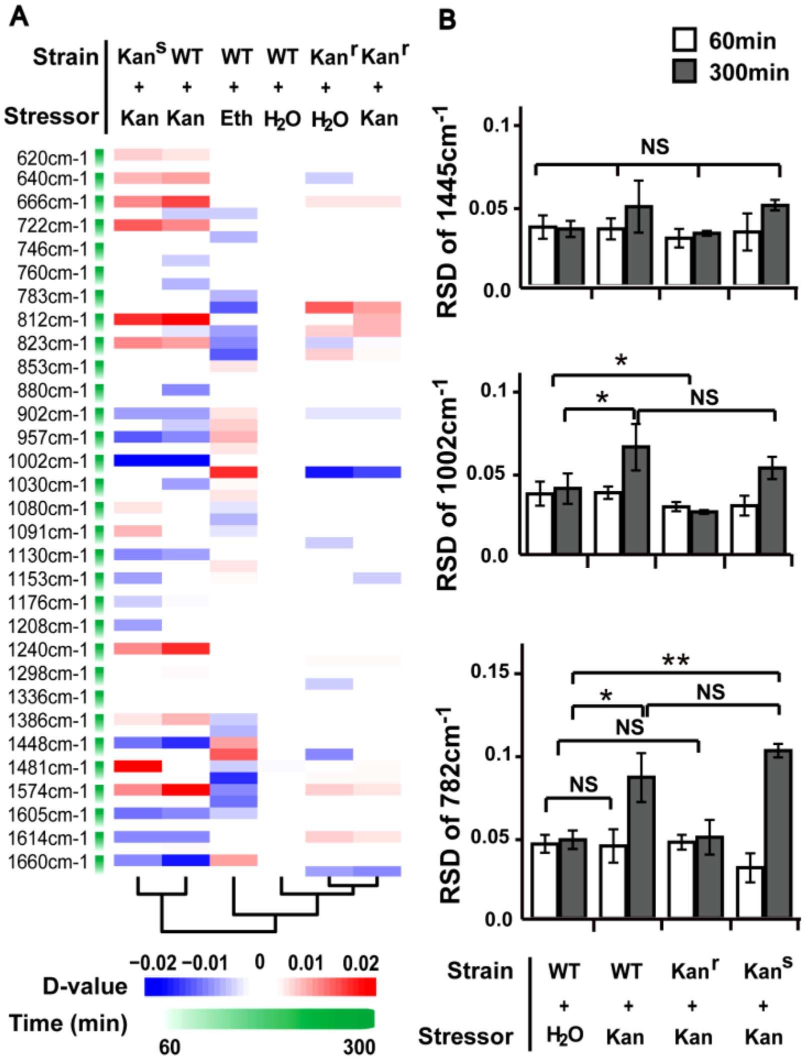

REFERENCES

(1) Wang, Y.; Ji, Y. T.; Wharfe, E. S.; Meadows, R. S.; March, P.; Goodacre, R.; Xu, J.; Huang, W. E. *Anal Chem* **2013**, *85*, 10697-10701.

(2) Moritz, T. J.; Polage, C. R.; Taylor, D. S.; Krol, D. M.; Lane, S. M.; Chan, J. W. *J Clin Microbiol* **2010**, *48*, 4287-4290.

(3) Ji, Y.; He, Y.; Cui, Y.; Wang, T.; Wang, Y.; Li, Y.; Huang, W. E.; Xu, J. *Biotech J* **2014**, *9*, 1512-1518.

(4) Wang, T.; Ji, Y.; Wang, Y.; Jia, J.; Li, J.; Huang, S.; Han, D.; Hu, Q.; Huang, W. E.; Xu, J. *Biotechnol Biofuels* **2014**, *7*, 58.

(5) Moritz, T. J.; Taylor, D. S.; Polage, C. R.; Krol, D. M.; Lane, S. M.; Chan, J. W. *Anal Chem* **2010**, *82*, 2703-2710.

(6) Wold, S.; Martens, H.; Wold, H. *Lect Notes Math* **1983**, *973*, 286-293.

(7) Hu, P.; Borglin, S.; Kamennaya, N. A.; Chen, L.; Park, H.; Mahoney, L.; Kijac, A.; Shan, G.; Chavarria, K. L.; Zhang, C. M.; Quinn, N. W. T.; Wemmer, D.; Holman, H. Y.; Jansson, C. *Applied Energy* **2013**, *102*, 850-859.

(8) Alfassi, Z. B. *J Am Soc Mass Spectr* **2004**, *15*, 385-387.

(9) Breiman, L. *Mach Learn* **2001**, *45*, 5-32.

(10) Zweig, M. H.; Campbell, G. *Clin Chem* **1993**, *39*, 1589-1589.
